# Forecasting habitat suitability of tropical karst plants in a warmer world — Thailand’s *Begonia* diversity as a key example

**DOI:** 10.3389/fpls.2025.1496040

**Published:** 2025-05-01

**Authors:** Sirilak Radbouchoom, Marjorie D. delos Angeles, Boniface K. Ngarega, Thamarat Phutthai, Harald Schneider

**Affiliations:** ^1^ Center for Integrative Conservation & Yunnan Key Laboratory for Conservation of Tropical Rainforests and Asian Elephants, Xishuangbanna Tropical Botanical Garden, Chinese Academy of Sciences, Menglun, Yunnan, China; ^2^ University of Chinese Academy of Sciences, Beijing, China; ^3^ Plant Biology Division, Institute of Biological Sciences, College of Arts and Sciences, University of the Philippines Los Baños, Los Baños, Philippines; ^4^ Department of Botany, Jomo Kenyatta University of Agriculture and Technology, Nairobi, Kenya; ^5^ Faculty of Environment and Resource Studies, Mahidol University, Nakhon Pathom, Thailand

**Keywords:** climate change, conservation, ensemble model, habitat preferences, plant species with extremely small populations, species distribution model

## Abstract

Tropical karst habitats host a rich plant diversity, of which many species are edaphic specialists with narrow distribution ranges. Many of these plants are expected to be highly vulnerable to global climate change as a consequence of the substantial fragmentation of karst formations in combination with edaphic preferences and dispersal limitations. In recent years, the application of species distribution models to predict range under future climate scenarios has increasingly become a popular tool to guide conservation management approaches. Here, we examined the impact of climate change on the genus *Begonia* in Thailand using an ensemble modelling approach. The models incorporated climatic data and the geological characteristics of karst formations to reliably predict the distribution of species that reside within karst habitats. Our results revealed that the diversity of *Begonia* species in karst environments is primarily influenced by key climatic factors, including the mean temperature of the wettest quarter and annual precipitation, along with geographical features such as karst formations. Together, these elements significantly shape the distribution patterns of *Begonia* diversity in these unique habitats. Under current climatic conditions, clusters of suitable habitats for *Begonia* were found in Northern, South-Western, and Southern Thailand. The employed scenarios for future warmer climates converged to predict a substantial loss of currently suitable habitats. Applying the moderate SSP245 scenario, the model predicted range losses of 32.46% in 2050 that accumulate to 38.55% in 2070. Notably, more worrying predictions were obtained by applying the worst-case (SSP585) scenario, which projected a range loss of 37.73% in 2050 and increasing to 62.81% in 2070. In turn, the gain by areas becoming suitable was much lower than the loss. These results are highly consistent with the predicted high vulnerability of karst plants to global climatic change. Conservation efforts require taking into account these predictions by focusing on two key actions. Firstly, protecting areas where occurrences of *Begonia* are predicted to be less affected by climate change. The assignment of these areas to national parks thus far has not been achieved yet. Secondly, establishing practical conservation strategies for *Begonia* species occurring preliminary or even exclusively in karst landscapes.

## 1 Introduction

Karst formations formed on soluble rocks such as limestone and marble (Clements et al., 2006) are known to be home to a diverse range of ecological specialists and consequently many endemic species with extremely small populations (Clements et al., 2006; Geekiyanage et al., 2019; Hughes, 2017). Furthermore, karst habitats are key examples of the growing body of evidence supporting the crucial role of edaphic properties, alongside climatic conditions, in shaping the spatio-temporal patterns of plant diversity (Rajakaruna, 2004, 2018; Hulshof and Spasojevic, 2020). In turn, karst ecosystems are considered extremely fragile ecosystems that are highly vulnerable not only to loss of habitat as a consequence of mining activities and other human- or natural-induces calamities (Veni et al., 2001; Zhao and Hou, 2019; Zhang et al., 2021). In particular, tropical karst habitats have arguably not received the urgently required attention in conservation efforts (Xiao and Weng, 2007; Zhu et al., 2017; Hughes, 2017). The diverse microhabitats of karst ecosystems foster a high degree of species endemism (Du et al., 2017; Meng et al., 2024). Many plant species found in karst landscapes have small populations and often exhibit highly fragmented distribution ranges as a consequence of the topographic structuring of karst formations (Du et al., 2017; Hu et al., 2023). Plant species that occur preferably or exclusively in karst habitats are expected to be especially vulnerable to global climate change because of the high fragmentation of karst landscapes (Enquist et al., 2019; Hannah et al., 2020; Urban, 2015). Monitoring the plant diversity of these habitats is crucial to achieving the target of zero plant extinction (Corlett and Tomlinson, 2020; Geekiyanage et al., 2019; Hulshof and Spasojevic, 2020; Radbouchoom et al., 2024), which requires substantial improvement of the quantity and quality of species records as well as the analytical procedures to be utilized to predict the extinction threats under current and future climatic conditions which are in turn urgently needed to prioritize conservation areas and measurements to protect these threatened plant species and their vulnerable habitats (Xu et al., 2021).

Species Distribution Modelling (SDM) are now widely utilized to predict the distribution ranges of plant species by focusing on the relationship between species and abiotic environment factors by focusing on the Hutchinsonian niche occupation (Guisan and Thuiller, 2005). Therefore, SDMs are expected to enhance our capacity to elucidate species distribution patterns and their responses to current and future environmental changes with climate data as predictors (Elith and Leathwick, 2009; Wiens et al., 2009) and are subsequently employed in a wide range of applications (Pearson, 2010), including the establishment of conservation management plans targeting threatened species or vulnerable habitats (Wang et al., 2016; Spiers et al., 2018; Chavy et al., 2019; Sofaer et al., 2019; Supsup et al., 2023; Stas et al., 2020; Parveen et al., 2022), biodiversity assessment predicting future species’ distributions under climate change (Liu et al., 2021; Soilhi et al., 2022), and management of invasive species (Panda and Behera, 2019; Zhang et al., 2021). Whereas the vast majority of studies employing SDMs still used exclusively or predominantly climatic factors, the incorporation of edaphic data into plant species distribution models has proven to improve the predicting power of SDMs for plant species (Beauregard and de Blois, 2014; Bertrand et al., 2012; Roe et al., 2022; Obico et al., 2023). The improved accuracy achieved by including both climatic and edaphic predictor variables is particularly important for studies focusing on plant species with limited geographic ranges at finer spatial scales (Austin and Van Niel, 2011).

The mega-diverse genus *Begonia* L. comprises about 2,151 species distributed across tropical and subtropical regions of the world, with the exception of Australia where this genus is missing (Hughes et al., 2015; http://padme.rbge.org.uk/Begonia). Begonias can thrive in a wide range of habitat types, while the occurrence of the majority species is arguably restricted to a narrow geographical range as a consequence of highly specialized environmental preferences in particular adaptation to edaphic conditions (Chung et al., 2014; Goodall-Copestake et al., 2009; Kiew, 1998; Kiew et al., 2015; Phutthai et al., 2009, 2014, 2019). Therefore, many species belonging to this genus are expected to be highly vulnerable to extinction threats due to their ecological specialization (narrow Hutchinsonian niche occupation) and often extremely small population size (Pimm, 2021). Conversely, plant species with a small distribution range are often overlooked, resulting in a general trend to underestimate their contribution to the regional or global species diversity. To overcome these challenges, it is necessary to enhance our efforts to document the spatial distribution of these plants, their ecological preferences, and population sizes through enhanced research efforts. Improving our understanding of the influence of edaphic factors such as karst landscapes on *Begonia* distribution patterns is urgently needed but hampered by the restricted efforts to study the ecological preferences of these plants.

The *Begonia* diversity of Thailand is arguably an outstanding example of addressing this research gap because this diversity is extremely well-documented as a consequence of research efforts in recent years. Currently, 61 species of *Begonia* have been reported to occur in Thailand (Hughes et al., 2015; Phutthai et al., 2019; Radbouchoom et al., 2023), of which 40% are considered country endemics. In the context of this study, over 70% of Thailand’s *Begonia* occurs on karst formations (Phutthai et al., 2009). Utilizing the well studied *Begonia* diversity of Thailand as a case study, this study aims to elucidate the role of the edaphic preferences to karst formations in the context of their vulnerability to extinction threats, especially to anthropogenic-induced climate change. By doing so, we established clear evidence for using *Begonia* as a case study to investigate and forecast the future of high-habitat preference plants in karst regions by employing an ensemble distribution model to determine the environmental factors that affect the distribution of *Begonia*. Furthermore, through future forecasting predictions, we were able to assess preliminary conservation efforts for *Begonia* species. As a consequence, this study highlights the importance of understanding the impact of karst landscapes as a meaningful factor limiting the distribution range of *Begonia* under current and future climate scenarios. In particular, this study aimed to: 1) identify the environmental variables that shape the *Begonia* diversity occurring on karst formations in Thailand; 2) predict the current and future distributions of *Begonia* under two CMIP6, the socioeconomic narratives or “Shared Socioeconomic Pathways” (SSP245 and SSP585) for the 2050s and 2070s; and 3) identify high-priority conservation areas and enhance the understanding of the ecological adaptations, ranges, and conservation status of the diverse genus *Begonia*.

## 2 Materials and methods

### 2.1 Study area

Thailand is situated in the Asia-Tropical vascular plant diversity dark spot because of the Linnean and Wallace shortfalls to document the rich diversity of this area (Ondo et al., 2024). More specifically, Thailand is part of the Indochinese subdivision of Southeast Asia, with the Peninsula forming a bridge between Indochina and the Malay Archipelago (de Bruyn et al., 2014). Thailand boasts a rich flora of approximately 12,500 known plant species (Trisurat et al., 2011). Considering the known plant diversity, the ongoing Flora of Thailand project has divided the phytogeography of Thailand into seven distinct regions: Northern, North-Eastern, Eastern, South-Western, Central, South-Eastern, and Peninsular (**Supplementary Figure S1**; Middleton, 2003; Phutthai et al., 2019). The country hosts a notable uniqueness with approximately 800 endemic plant species. Plant diversity assessment of karst formation in Thailand reported about 180 species (22.5%) occurring exclusively in limestone karst areas (Santisuk et al., 2006).

### 2.2 Collection of occurrence data

A total of 2,681 voucher specimens from 61 *Begonia* species occurring in Thailand were compiled through several data collection processes. First, data were gathered from voucher specimens deposited in various herbaria, including AAU, ABD, BM, BK, BKF, E, K, KUN, L, P, PE, QBG, and SING (**Supplementary Table S1**). Physical examination of vouchers was conducted at the AAU, ABD, BKF, BK, BM, E, K, L, PSU, and SING herbaria by TP, with updated information from BK, BKF, and QBG provided by SR. Second, occurrence data were also obtained through the field survey observation from citizen science. While this data source has the potential to improve the precision of spatial occurrences used in the analysis, it introduced some taxonomic uncertainties, as species identification was not always verified by taxon experts. Third, occurrence data were sourced from online databases, including the Begonia Resource Center (https://padme.rbge.org.uk/Begonia/home; Hughes et al., 2015), Global Biodiversity Information Facility (GBIF: https://www.gbif.org) and iNaturalist (https://www.inaturalist.org). Finally, data from relevant publications and field surveys were incorporated (Peng et al., 2017; Phutthai et al., 2009, 2012, 2014, 2019, 2021; Phutthai and Hughes, 2016, 2017a, b; Phutthai and Sridith, 2010; Radbouchoom et al., 2023). Erroneous coordinates obtained via human observation, botanic gardens, and unreasonable sites were excluded from the analysis. The majority of occurrences were selected based on the available GPS data from several sources to ensure the accuracy of the data points. In cases of species lacking GPS records, we assigned the nearest location indicated on the specimen labels. Additionally, all species identifications used in this study were checked and updated by SR and TP according to the currently accepted names (Hughes, 2008; Moonlight et al., 2018).

The number of occurrences per species was limited for many species as a consequence of their small distribution ranges and ecological specialization, which challenged access to these locations. This characteristic presents significant challenges in creating species distribution models (SDMs) that reliably predict species distributions (Thomas et al., 2024), given that only seven species have over ten occurrences available from GPS records following the data cleaning process. Furthermore, studies using species-level models tend to ignore the contribution of local adaptive responses by assuming that a species’ present distribution reflects the entire range of suitable conditions, meaning the fundamental niche versus the realized niche (Smith et al., 2019). To address this limitation, we implemented a lumping strategy, pooling the presence records of the 41 selected species with shared habitat preference in karst formation into a single class. In this study, species with shared habitat preferences serve as surrogates for other species with similar ecological conditions but insufficient occurrence data (Ferrier, 2002; Ferrier and Guisan, 2006). To ensure the inclusion of species with shared habitat preferences, we established the following criteria for selection: i) Herbarium specimens labelled with specific occurrences in karst regions, supported by publications and field surveys. ii) A spatial overlay of *Begonia* occurrence records with a karst formation map, confirming their distribution in these areas. iii) Expert evaluation to validate the presence of *Begonia* species in karst landform. The results were thus interpreted as representative of *Begonia* species with a high preference for karst habitats rather than a representation of the genus.

### 2.3 Data preparation

Occurrence records were filtered by thinning duplicate records within 1 km^2^ using “*spThin*” package in R version 4.3.0 (Aiello-Lammens et al., 2015; R Core Team, 2023). After the cleaning process, a total of 214 occurrences from 41 *Begonia* species were used in analyses (**Supplementary Tables S2, S3**). These records were utilized to generate SDMs that identify the probability of habitat suitability for *Begonia* under the current climate conditions. Two datasets were utilized to relate the current presence of *Begonia* species to various environmental factors by combining different predictors that affect the model’s performance (Velazco et al., 2017). The first dataset comprised 19 bioclimatic variables that were obtained from the WorldClim database version 2.1 (https://www.worldclim.org/data/worldclim21.html) (Fick and Hijmans, 2017), which covered the average climatic conditions for the years 1970-2000. The second data set consisted of the world karst aquifer map, a categorical environmental variable obtained from the World-wide Hydrogeological Mapping and Assessment Programme (WHYMAP) (https://www.whymap.org; BGR, 2016). The nineteen bioclimatic variables, along with the geological data (karst landform) were tested for multicollinearity using Pearson’s correlation coefficient (r > 0.70) with “raster” and “terra” packages in R (Fournier et al., 2017; Brun et al., 2020; de Lima et al., 2022). Furthermore, potential multicollinearity issues were addressed using the “usdm” package in R (Naimi, 2017), and variables with a VIF greater than 10 were excluded (**Supplementary Figure S2**; **Supplementary Table S4**). The final environmental factors were selected based on their ecological relevance and ability to enhance model performance. This process resulted in the inclusion of six environmental variables, namely: mean diurnal range (BIO2), mean temperature of wettest quarter (BIO8), annual precipitation (BIO12), precipitation seasonality (BIO15), precipitation of warmest quarter (BIO18) and karst landform.

### 2.4 Species distribution model

Species distribution models were generated using ensemble model approach as implemented in the “*biomod2*” package in R version 4.3.0 (Thuiller et al., 2009; 2024; R Core Team, 2023). The ensemble model approach was employed by considering its potential to enhance the accuracy of the model prediction as compared to a single-model approach (Araújo and New, 2006; Harris et al., 2023). The employed ensemble modelling approach incorporated five statistical approaches, namely Classification Tree Analysis (CTA), Generalized Additive Model (GAM), Generalized Boosting Model (GBM), Random Forests (RF), eXtreme Gradient Boosting Training (XGBOOST).

Absence data were generated using pseudo-absence points, with a ratio of five to one compared to the presence points. A total of 45 models were generated, resulting from 3 iterations of models, 3 sets of pseudo-absences, and five unique models (de Lima et al., 2022). The data was split randomly into calibration (80%) and evaluation (20%) for each model iteration and pseudo-absence set. Following the arguments of Konowalik and Nosol (2021), the performance of each model and ensemble model was assessed by calculating the area under the receiver operating characteristic (ROC) curve (AUC) and true skill statistic (TSS) (Hanley and McNeil, 1982; Allouche et al., 2006). The model ensembles were required to meet a threshold of AUC ≥ 0.9 (with a range of 0-1, where 1 is considered the best and 0.5 is as good as random) and TSS ≥ 0.7 (with a range of -1 to 1, where 1 indicates perfect agreement and values less than 1 indicate less than perfect agreement). The variable importance analysis was conducted using six iterations, which yielded importance scores ranging from 0 (no impact) to 1 (highest importance) (Stankovic et al., 2022). The results were presented as average values and their standard deviation (Average ± SD). Response curves were generated for the most important predictors identified by the ensemble models to enhance the evaluation of how each species responds to the environmental space. These curves depict the relationship between the probability of occurrence and one predictor variable while averaging the effects for each unique prediction value. The probability of habitat suitability maps was divided into four levels of potentially suitable areas according to the predicted probabilities of presence between 0 and 1, unsuitable habitats (0.0–0.2), habitats of low suitability (0.2–0.4), habitats of medium suitability (0.4–0.6), and habitats of high suitability (0.6–1.0) (Xiao et al., 2022).

Ensemble forecasting of species distribution model was employed to evaluate the vulnerability of plant species to climate change resulting from anthropogenic activities. The analysis was conducted for future climate scenarios in 2050 and 2070, considering two distinct socioeconomic narratives or Shared Socioeconomic Pathways (SSP). The two scenarios were based on the Coupled Model Intercomparison Project (CMIP6). The Intergovernmental Panel on Climate Change (IPCC) recommended the Shared Socio-Economic Pathways (SSPs) as a means of modelling future climate change because this approach was found to be more robust than the four Representative Concentration Pathways (RCPs) utilized in AR5 (IPCC, 2023). Conservation decisions based on CMIP6 projection were considered to have a higher degree of confidence in the Southeast Asian region (Hamed et al., 2022). This study explored the climatic conditions, specifically focusing on SSP245, and SSP585. These scenarios were determined based on socioeconomic factors and greenhouse gas emissions projections for the year 2100, with SSP245 representing medium emissions or middle of the road scenario (4.5 W/m²) and SSP585 representing high emissions or worst-case scenario (8.5 W/m²) (IPCC, 2023). The climate scenarios were obtained from the Model for Interdisciplinary Research on Climate v6 (MIROC6; Tatebe et al., 2019) and included 19 bioclimatic variables (BIO1–BIO19) (**Supplementary Table S5**). The environmental variables were clipped based on Thailand’s extent and projected using CSR (WGS84). Afterwards, they were resampled at a spatial resolution of 30 arc seconds (~1 km^2^) for each period using QGIS version 3.28.2 and the “Raster” package in R version 4.3.0 (Hijmans, 2023; QGIS.org, 2021; R Core Team, 2023).

## 3 Results

### 3.1 Habitat suitability and the current and warmer global climates

According to the assessed model performance, Generalized Boosting Model (GBM) model exhibited the best performance followed by Random Forests (RF) and eXtreme Gradient Boosting Training (XGBOOST) (**Table 1**). In general, the ensemble models yielded high values for AUC (0.94), TSS (0.71), sensitivity (93.06), and specificity (77.50), indicating the reliability of the prediction results (see **Table 1**). The most influential abiotic factors shaping the distribution of these *Begonia* species were identified as mean temperature of wettest quarter (BIO8: 48.92%), followed by annual precipitation (BIO12: 22.70%) and karst landform (9.77%). Other variables such as precipitation of warmest quarter (BIO18: 7.4%), precipitation seasonality (BIO15: 5.62%) and mean diurnal range (BIO2: 5.59%) contributed less (**Table 2**). The relationship between bioclimatic and habitat variables affecting the current distribution of Thailand’s karst *Begonia* species was detected in the ecological thresholds for the main contributing environmental factors, namely mean temperature of wettest quarter (BIO8: 18.42-28.6°C/quarter), annual precipitation (BIO12: 781-4,302 mm/month), karst landform, precipitation of warmest quarter (BIO18: 154-1,850 mm/quarter), precipitation seasonality (BIO15: 35.78-108.7) and mean diurnal range (BIO2: 6.63-12.77°C) (**Table 2**; **Supplementary Figure S3**).

**Table 1:** Mean validation scores of the methods utilized to generate species distribution models for Thailand’s *Begonia* species found on karst formation.

Models	AUC (mean ± SD)	TSS (mean ± SD)	Sensitivity (mean ± SD)	Specificity (mean ± SD)
Generalized Boosting Model (GBM)	0.99 ± 0.00	0.98 ± 0.01	87.40	98.16
Random Forests (RF)	0.978± 0.00	0.89 ± 0.01	98.52	90.63
eXtreme Gradient Boosting Training (XGBOOST)	0.98 ± 0.00	0.86 ± 0.01	95.98	89.98
Classification Tree Analysis (CTA)	0.82 ± 0.03	0.59 ± 0.05	88.57	70.45
Generalized Additive Model (GAM)	0.86 ± 0.01	0.61 ± 0.02	87.40	73.36
Ensemble model	0.94 ± 0.01	0.71 ± 0.03	93.06	77.50

Reported statistics: AUC, Area under the receiver operating characteristic curve; TSS, True skill statistic scores based on single and ensemble models; Sensitivity; and Specificity. each value was given as a mean plus standard deviation. In total, five statistical approaches (CTA, GAM, GBM, RF and XGBOOST) plus one ensemble model were recorded.

**Table 2:** Estimated contribution and suitable threshold of the six abiotic environmental factors to the predicted occurrence of Thailand’s karst variable importance of *Begonia* species.

Environmental variable	Variable relative contribution (%)	Suitable threshold
Mean Temperature of Wettest Quarter (BIO8)	48.92	18.42-28.6°C
Annual precipitation (BIO12)	22.70	781-4,302 mm
Karst landform	9.77	
Precipitation of warmest quarter (BIO18)	7.4	154-1,850 mm
Precipitation Seasonality (Coefficient of Variation) (BIO15)	5.62	35.78-108.7
Mean Diurnal Range [Mean of monthly (max temp - min temp)] (BIO2)	5.59	6.63-12.77°C

### 3.2 Models’ performance and variable contribution

**Figure 1:**
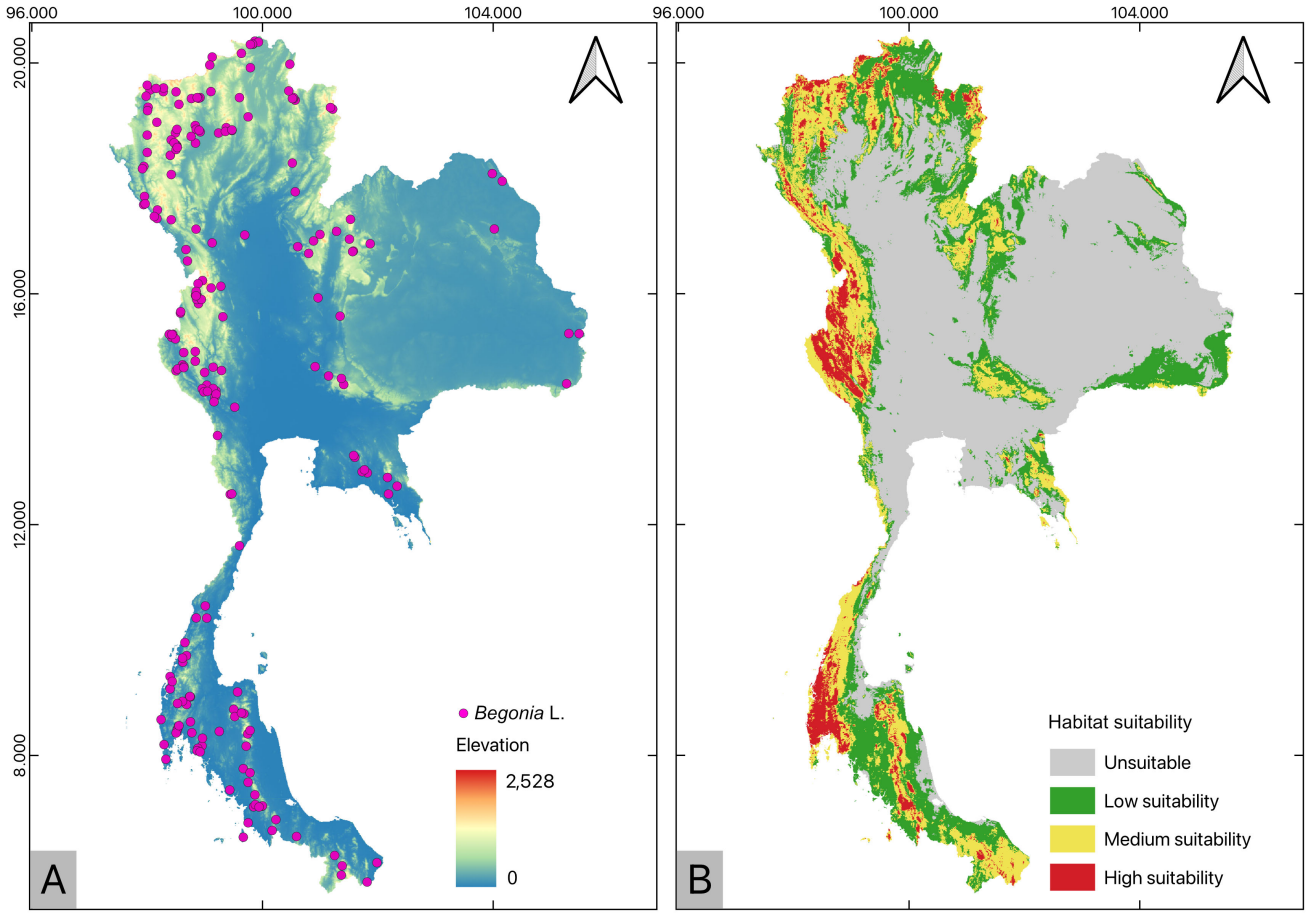
Recorded distribution and predicted distribution of Thailand’s karst *Begonia* species under the current climatic condition using an ensemble model. **(A)** Recorded distribution of Thailand’s karst *Begonia* species visualized against the elevation distribution. **(B)** Predicted distribution of habitats with high, medium, low suitability and unsuitable as produced in the ensemble model for the current climatic conditions.

Occurrence records (**Figures 1A, B**) and distribution predictions revealed that Thailand’s karst *Begonia* species occurred primarily along continuous mountain ranges in Northern and South-Western Thailand, and Peninsular region. Scattered suitable habitats were predicted to occur in North-Eastern, Central, and South-Eastern Thailand. The total predicted suitable habitat area spanned 224,594 km^2^, with the Northern region boasting the highest concentration of highly suitable habitats in Chiang Mai, Chiang Rai, Lampang, Lamphun, Mae Hong Son, Tak, Prayao, Prae, and Nan. In the South-Western region, highly suitable habitats were predominantly found in Kanchanaburi, Tak, and Uthai Thani. Notably, Thung Yai Naresuan, Um Phang, and Khao Laem exhibit the greatest habitat suitability within designated protected areas. In Northern Thailand, significant protected sites included Hui Nam Dang, Doi Phu Ka, and Lum Nam Pai, while in the Southern, Khao Banthat, Khao Sok, and Khlong Saeng were identified as highly suitable habitat areas. These protected areas have been recognized as important conservation areas for undiscovered species and may serve as critical refuges for *Begonia* in the future (**Supplementary Table S6**). In contrast, Eastern and Central Thailand were dominated by a large area of low suitability, covering 113,082 km^2^.

**Figure 2:**
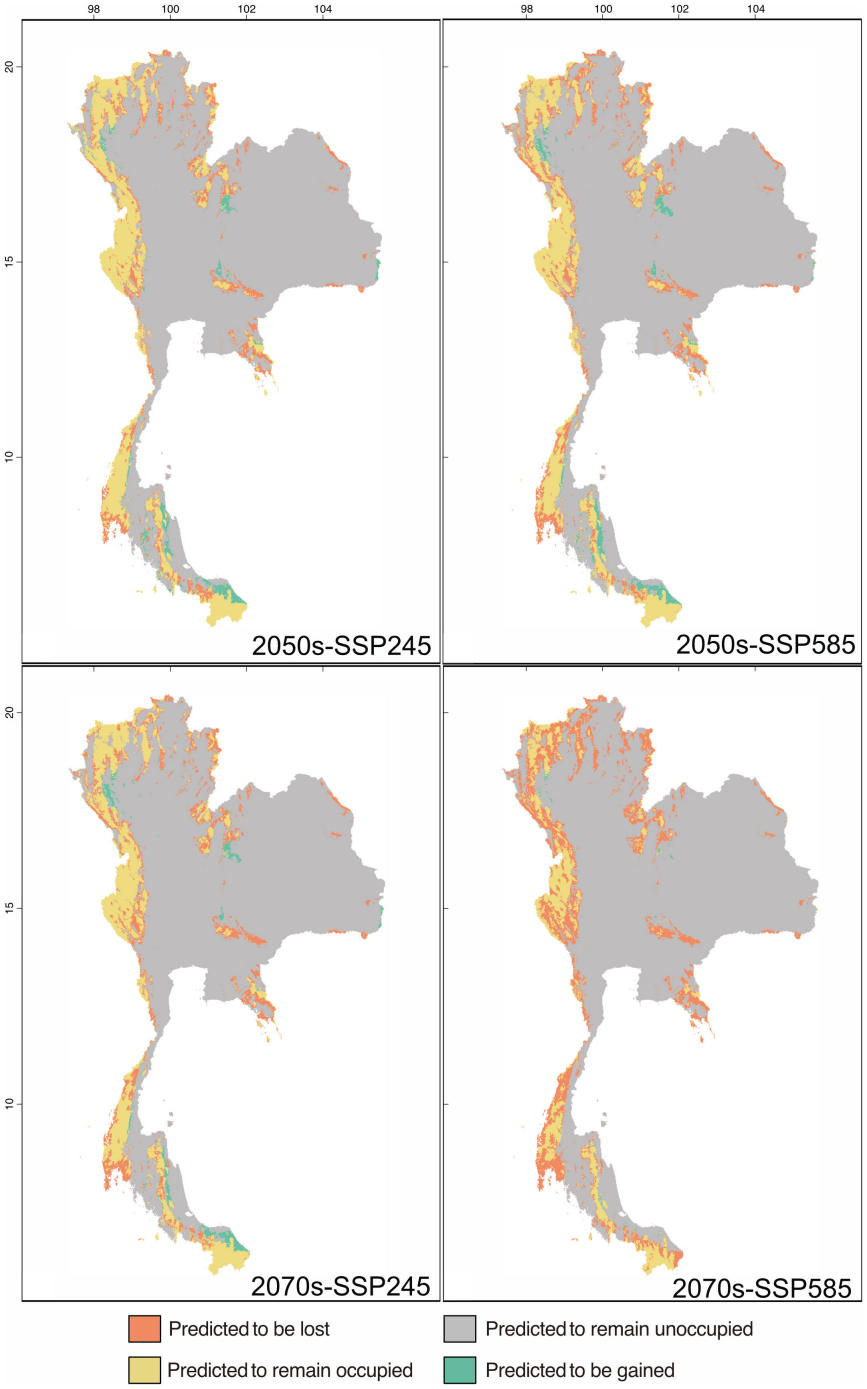
The distribution of *Begonia* in Thailand across different periods and climate scenarios. The visualization illustrates the projected changes, indicating areas predicted to be lost (orange), gained (green), remain occupied (yellow), and unoccupied (grey) as determined by the model.

**Figure 3:**
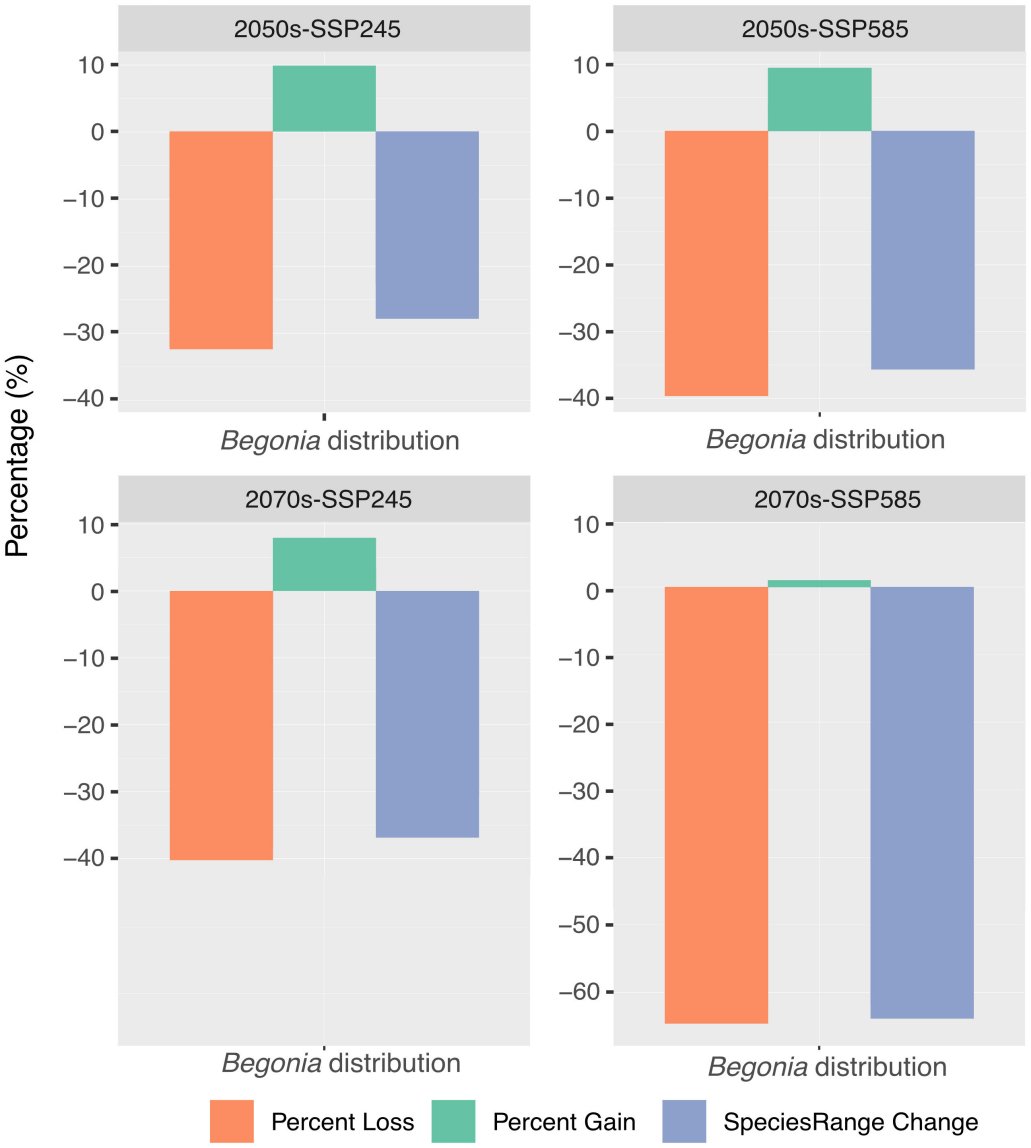
The potential future distribution of suitable areas for *Begonia* in the 2050s and 2070s under shared socioeconomic pathway (SSP) scenarios. The outcomes for two different SSP scenarios are represented by the medium greenhouse gas emissions concentration (SSP245) and the high greenhouse gas emissions concentration (SSP585). The color codes in the bar chart indicate the percentage of areas loss, gain and species range change.

The predictive models of the distribution of suitable habitats for Thailand’s karst *Begonia* species by utilizing SSP245 and SSP585 for the years 2050s and 2070s identified stable (no change) areas spanning from 51,044 km^2^ to 92,694 km^2^, the percentage of area loss ranging from 32.46% to 68.81%, and area gain ranging from 0.98% to 9.82% (**Table 3**). The majority of the suitable habitat areas along the border of Northern, South-Western, and Peninsular Thailand were predicted to undergo range shift, especially in the year 2070s under both scenarios (**Table 3**; **Supplementary Figures S4–S6**). The stable areas in SSP245 were projected to decrease significantly over the next few decades (**Figures 2**, **3**). By the 2050s, these areas were expected to be restricted to only 92,694 km^2^. While there were some positive predictions with 9.82% gain offsetting 32.46% decline in habitat suitability, these findings suggested that there was -22.64% total range change. The situation worsened in the 2070s model, with stable areas decreased to 84,341 km^2^ and habitat suitability experienced 38.55% loss and only 7.90% gain. The overall total range change observed was expected to be -30.65%. Meanwhile, the habitat suitability area of *Begonia* expected to get worse in SSP585 with the high emission of greenhouse gases. By the 2050s, the stable areas measured 85,471 km^2^, with 37.73% loss in habitat suitability and 9.43% gain. The total range change was -28.29%. Fast forward to the 2070s, and the stable areas had decreased to 51,044 km^2^, with a significant 62.81% loss in habitat suitability, the highest among different periods and scenarios. However, there was only 0.98% gain, resulting in a total range change of -61.83%. These findings indicated major threats to Thailand’s *Begonia* diversity as a consequence of global warming enforced major shifts in the suitable habitat ranges (**Table 3**).

**Table 3:** Summary of species range change statistics.

	2050s-SSP245	2070s-SSP245	2050s-SSP585	2070s-SSP585
Loss	44,556	52,909	51,779	86,206
Stable0/No Occupancy	473,199	475,838	473,735	485,340
Stable1/No Change	92,694	84,341	85,471	51,044
Gain	13,484	10,845	12,948	1,343
Percent Loss	32.46	38.55	37.73	62.81
Percent Gain	9.82	7.90	9.43	0.98
Species Range Change	-22.64	-30.65	-28.29	-61.83
Current Range Size	137,250	137,250	137,250	137,250
Future Range Size0/No migration	92,694	84,341	85,471	51,044
Future Range Size1/Migration	106,178	95,186	98,419	52,387

Stable/No Occupancy represents the count of pixels currently unoccupied and not expected to be occupied in the future, whereas Stable/No Change represents the count of pixels that are presently occupied and are predicted to remain occupied in the future.

## 4 Discussions

### 4.1 The current habitat suitability and the effect of environmental variables on *Begonia* diversity in Thailand

This study marks the first investigation into utilizing karst landform to forecast suitable habitats for *Begonia* species by generating species distribution models under current and future climate scenarios. The results demonstrate that both climatic and geographical factors significantly influence the distribution of *Begonia*. Among these factors, temperature emerged as the most influential, consistent with its role as a critical determinant of plant distributions globally (Moles et al., 2009). Temperature fluctuations have profound impacts on plant physiology and ecological interactions, shaping global plant distributions (Kerbler and Wigge, 2023). Regions with higher temperature variability tend to support distinct plant groups (Huang et al., 2021). Even small shifts in temperature can have disproportionate impacts on tropical plant species compared to substantial changes in temperate zones (Doughty et al., 2023). These findings underscore the importance of temperature as a pivotal abiotic factor influencing *Begonia* distribution. In addition to temperature, annual precipitation (BIO12) was identified as another critical variable affecting the distribution of *Begonia* species. This aligns with previous studies indicating that *Begonia* species exhibit strong edaphic specialization and are significantly influenced by interactions between edaphic and climatic conditions (Phutthai et al., 2009; Thomas et al., 2012). Future climate scenarios predict changes in precipitation patterns, particularly in tropical regions (Afreen et al., 2019), which may further impact habitat suitability and may drive habitat loss or gain for *Begonia* populations in Thailand. Karst landforms emerged as another key factor shaping the distribution of selective species included in this model. Limestone karst regions in Southeast Asia provide optimal edaphic conditions and are renowned for their diverse *Begonia* flora (Cannon et al., 2009; Thomas et al., 2012). The unique microhabitats within these karst formations contribute to the species’ diversity, although the lack of comprehensive data on karst ecosystems presents a limitation in fully understanding their role. Further research into the interactions between *Begonia* species and their karst microhabitats is needed to refine our predictions and conservation strategies.

### 4.2 The potential distribution of *Begonia* in the future and range change

Climate change is one of the major factors contributing to changes in global biodiversity (Suggitt et al., 2023). Future projections indicate that this trend would continue, especially under medium to high greenhouse gas emission scenarios. These changes are expected to take place across various regions, with the most vulnerable habitats being in the Northern and South-Western regions, as well as Peninsular areas in Thailand. By 2070s, the reduction of potential suitability area was expected to be at its highest under the high greenhouse gas emission scenarios (SSP585). This loss was set to occur across the country, particularly in Northern and Western region. Under these conditions, many high suitability areas were expected to transition to medium suitability areas, resulting in net loss of habitat suitability for *Begonia* species. Under medium greenhouse gas emission scenarios (SSP245) in the 2050s, the potential suitability area is predicted to increase by approximately ten percent. However, *Begonia* experienced a range decrease due to the present loss of potential suitability area outweighing the gain. *Begonia* dispersed over long distances, resembling an archipelago-like pattern, this might make it even more challenging when facing the shift of suitable habitat (Thomas et al., 2012). This study highlighted the impact of climate change on edaphic species found in karst regions, revealing that the more greenhouse gas emissions, the greater the future loss of potential suitability areas. Nevertheless, this study also demonstrated the limitations of forecasting species distributions based on the currently available global climatic data and karst landform map. This data set has not integrated additional factors that may impact plant distribution, such as physiological tolerances, dispersal abilities, genetic diversity, and ecological interactions (Lee-Yaw et al., 2022), due to data availability constraints. Furthermore, conducting thorough ground surveys according to habitat suitability maps and incorporating other ecological factors would enhance our capacity to develop effective conservation strategies.

### 4.3 Conservation implications

Recent plant inventories conducted on the limestone karst have revealed limestone to be a biodiversity gem, with numerous species awaiting description (Gale et al., 2014; Phutthai et al., 2009, 2019; Puglisi et al., 2016; Radbouchoom et al., 2023; Sae Wai and Hu, 2020; Suddee et al., 2014; Triboun and Middleton, 2012). Notably, *Begonia* holds a significant position among the flourishing species of these regions, particularly in the karst region of the Malesia Peninsular, where it was regarded as a flagship species (Kiew, 1998; Nabila et al., 2011). We also highlighted the importance of considering plants with edaphically specialized plants in conservation planning efforts. These species were restricted to specific types of habitats and were therefore at risk of habitat loss and degradation. However, several of these vital habitat sites have not been fully documented, making them more susceptible to potential harm (Xu et al., 2021). In this study, we identified highly suitable habitats for *Begonia* in the karst region in each protected area in Thailand. In total, 80 protected areas hold the highest evaluation values consisting of high, medium, and low habitat suitability (**Supplementary Table S6**). Although protected areas in Thailand have been well-established, the potential suitable habitat for *Begonia* can also be found scattered outside these areas. It is imperative that we broaden our focus beyond protected areas and consider a new strategy to safeguard biodiversity and ensure the sustainability of our ecosystems effectively. When devising protection strategies for the future, it is imperative to take into account the impact of climate change on plant species, particularly those with specific habitat preferences and limited dispersal abilities. It is likely that plant species that have specific habitat preferences and limited ability to disperse, such as those that exist in karst substrates, are at a greater risk of extinction (Corlett and Tomlinson, 2020; Geekiyanage et al., 2019). The conservation of plant species with extremely small populations (PSESP) was a pressing issue in Thailand, given the rapid loss of habitat and degradation of ecosystems. Addressing practical conservation planning is essential to ensure the long-term viability of *Begonia* and its habitats. One of the key priorities is to identify local conservation areas capable of supporting the specific ecological requirements of *Begonia* with habitat preference on karst formation. Model predictions indicated that the total suitable habitat for *Begonia* is predominantly concentrated in the Northern, South-Western, and Southern regions of Thailand, where the highest habitat suitability was observed. Conversely, the Central and Eastern regions demonstrated lower habitat suitability due to limited ecological resources, extensive human land use, and less diverse topography such as karst landform. Notably, regions with high habitat suitability were closely associated with areas of elevated species richness, particularly in the South-Western, Northern, and Southern parts of the country (Radbouchoom et al., 2024). Despite Thailand’s well-established network of protected areas, conservation gaps persist. Plant species endemic to karst habitats have received inadequate attention. Thung Yai Naresuan, Um Phang and Khao Laem have been identified as critical conservation areas, exhibiting high habitat suitability for *Begonia* species inhabiting karst environments, with Thung Yai Naresuan Wildlife Sanctuary showing the highest suitability. However, suitable habitats in Northern and Southern Thailand should also be prioritized, as they support distinct *Begonia* diversity. Additionally, significant habitat suitability extends beyond protected areas, underscoring the need for targeted field surveys and strategic conservation planning to ensure the protection of these unprotected karst ecosystems.

### 4.4 Future research

This study acknowledged several limitations that merit consideration. A significant limitation is the accuracy and availability of GPS data for certain specimens. Many species lack precise geographic coordinates, and numerous historical specimens lack GPS information (Anderson et al., 2016). In this study, we minimized the bias by primarily utilizing most specimens with available GPS data, supplemented by human observation, and ensured data quality through expert validation by taxonomic specialists. We recommended that future research prioritize the collection of high-precision GPS data at collection sites, combined with detailed habitat documentation and photographic records. Another limitation raised from the inadequacy of the available maps of karst landforms and soil types, which lack sufficient detail. We have identified several species reported from karst regions; however, their occurrences were not reflected on the karst landform map. The application of remote sensing techniques may enable the generation of high-resolution, fine-scale karst maps (Qi et al., 2019; Theilen-Willige, 2024). While each karst region may have unique properties, the lack of extensively documented soil properties adds to another limitation. Furthermore, the inclusion of microclimatic data is essential to improve our understanding of how environmental conditions influence living organisms (Bramer et al., 2018; Marta et al., 2023). Future research direction could build upon the findings of this study to further advance our understanding of the ecological systems in this region. Another issue that urgently needs to be addressed is our limited understanding of the migration capacities of *Begonia* species. Improving or understanding the processes shaping the migration of *Begonia* species and the limited ranges observed for most species is essential to support conservation management. Given the fragmentation of karst landscapes, high dispersal capacities are expected to be needed to enable the plants to respond to climate change. However, alternative scenarios may consider much higher tolerances of the plant species to climate change than those predicted in this study.

## 5 Conclusion

While human activities are recognized as the major driver of plant diversity loss, our understanding of how plant species react to global climate change remains insufficient, especially in the context of species with edaphic specialization such as plants growing predominately or exclusively on karst formations. This study emphasized *Begonia* found in the karst formations of Thailand, highlighting the vulnerability of such species to anthropogenic climate change. Our findings reveal that the distribution of *Begonia* is heavily influenced by the often fragmented and topographic complex karst landform besides annual mean temperature and annual precipitation. Future projections recovered predictions of substantial shifts in habitat suitability for *Begonia* under the influence of medium (SSP245) and high (SSP585) greenhouse gas emissions scenarios. The escalation of greenhouse gas emissions is therefore expected to lead to a severe loss of suitable habitat for *Begonia* by the 2050s and 2070s and subsequently enhance the vulnerability of these species to other anthropomorphic extinction threats. To address these challenges, conservation strategies must consider the edaphic specialization of plants under current and future climatic conditions, with a particular focus on karst specialists restricted to specific habitats. Incorporating geoconservation in conservation strategies matters for all organisms (Gordon et al., 2021). Further comprehensive field surveys are essential to capture the full diversity of *Begonia* species and improve the data foundation to advance conservation planning. The additional approach included training programs for conservation officers to enhance their knowledge on prioritizing key species for effective monitoring and protection; engaging local communities through education and stewardship initiatives can further support conservation efforts, complemented by evaluating existing protected areas as climatic refuges for species with small populations. *In situ* conservation and habitat restoration efforts should focus on high-risk species with restricted ranges. Additionally, future research on plant communities in karst landforms can provide valuable insights into how to manage these unique ecosystems.

## Data Availability

The datasets presented in this study can be found in online repositories. The names of the repository/repositories and accession number(s) can be found in the article/**Supplementary Material**.
