# Supplementary material for: Forecasting habitat suitability of tropical karst plants in a warmer world — Thailand’s Begonia diversity as a key example
Source: Front Plant Sci. 2025 May 1;16:1496040. doi: 10.3389/fpls.2025.1496040 (PMC12078323; doi:10.3389/fpls.2025.1496040)
Supplement: Supplementary file 1 [file DataSheet1.docx]

**Supplementary file**


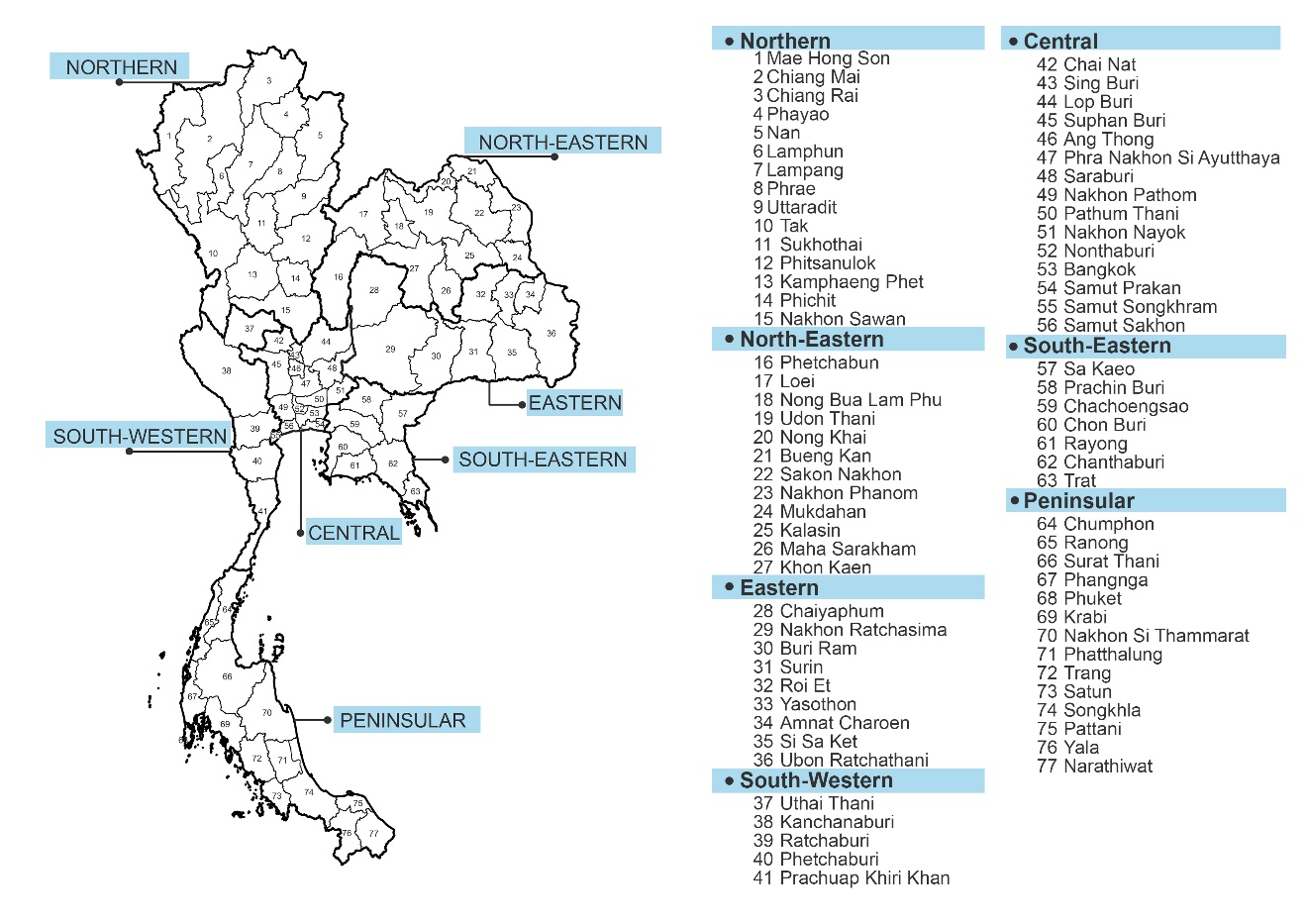


FIGURE S1. Floristic regions in Thailand (modified from flora of Thailand; map created in QGIS version 3.28.2)

FIGURE S2 Correlation matrix showing relationships between bioclimatic variables (BIO1 to BIO19) and Karst formation


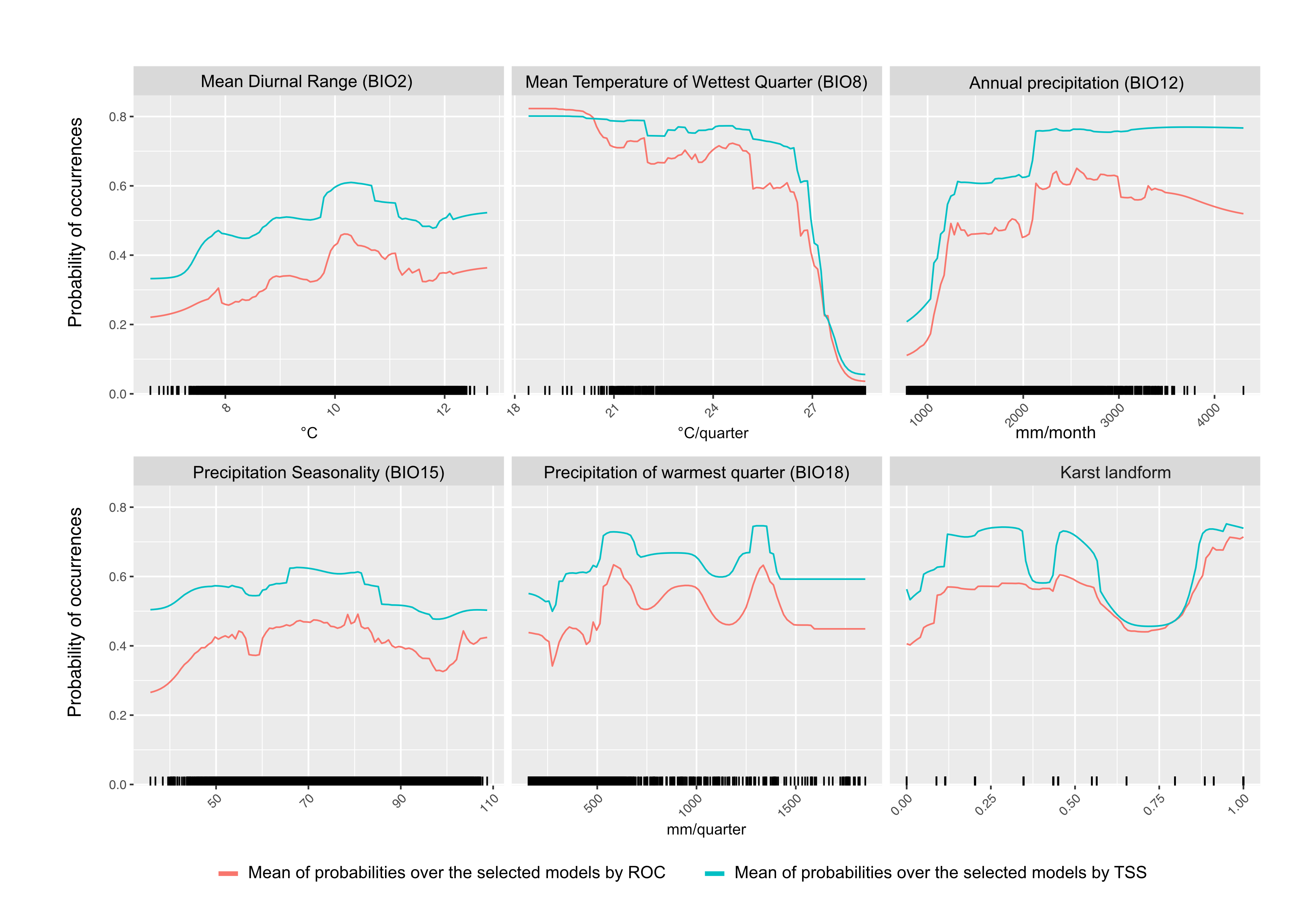


**FIGURE S3.** Response curves of the six most contributing environmental variables.


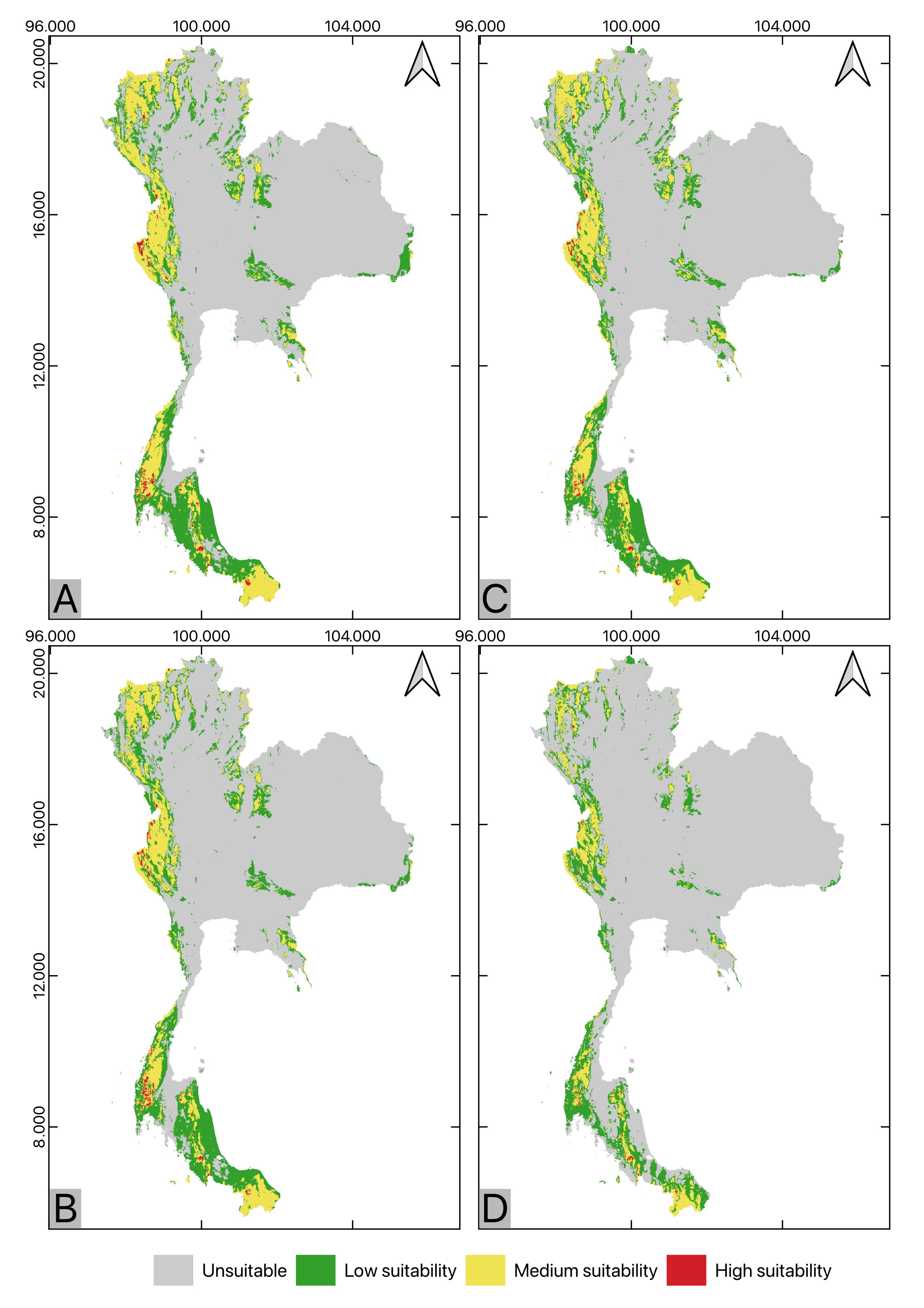


FIGURE S4 Predicted distribution of habitat suitability for Karst *Begonia* species under future climate scenarios. The maps displayed areas of high (red), medium (yellow), low (green), and unsuitable (white) habitat suitability. Predictions represented the years 2050 and 2070 under two Shared Socioeconomic Pathway (SSP) scenarios: SSP245 (medium greenhouse gas emissions concentration) and SSP585 (high greenhouse gas emissions concentration)


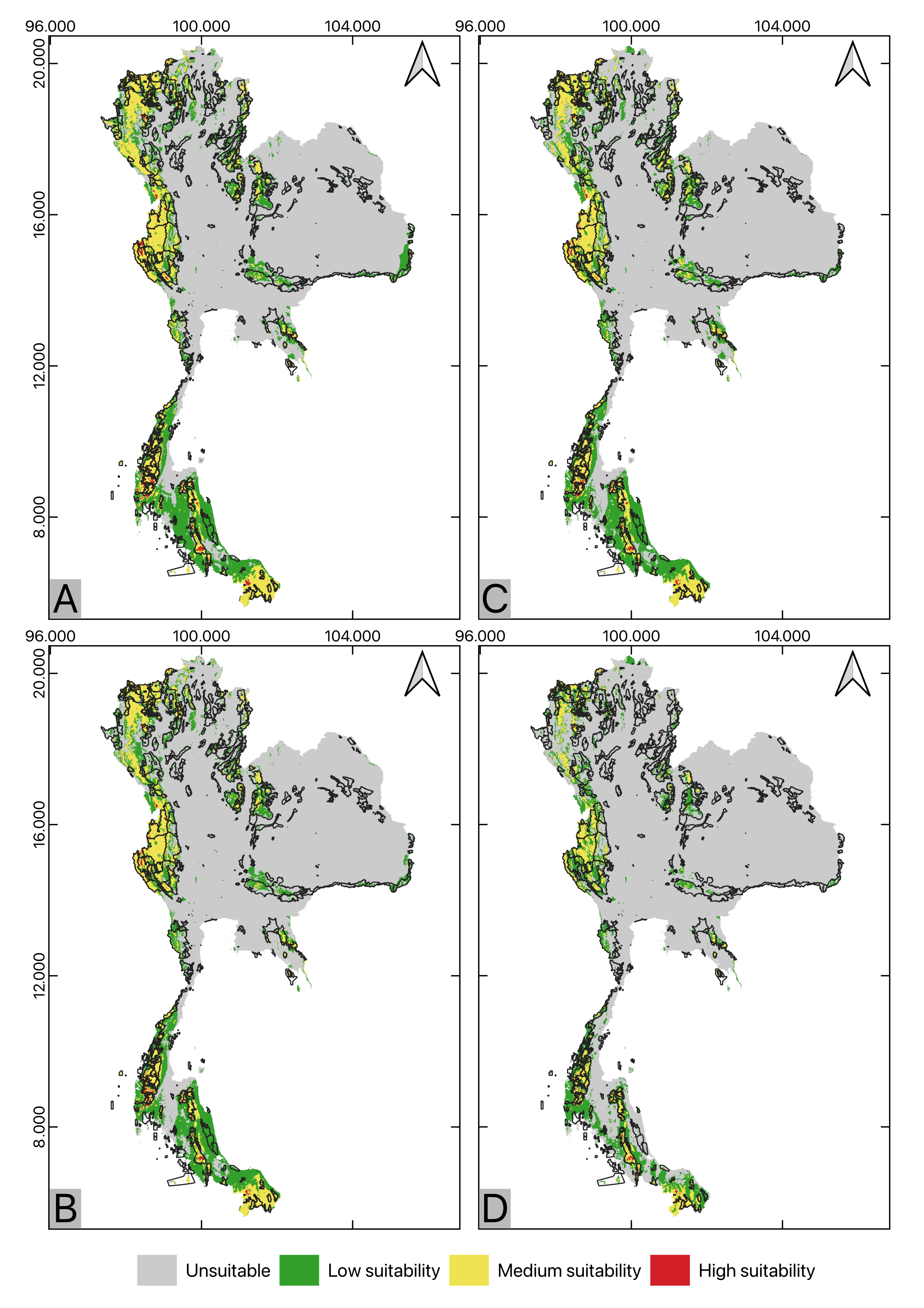


FIGURE S5 Predicted distribution of habitat suitability for Karst *Begonia* species under future climate scenarios, with protected areas overlaid. The maps shown regions of high (red), medium (yellow), low (green), and unsuitable (white) habitat suitability, with protected areas delineated as black boundaries. Predictions made for the years 2050 and 2070 under two Shared Socioeconomic Pathway (SSP) scenarios: SSP245 (medium greenhouse gas emissions concentration) and SSP585 (high greenhouse gas emissions concentration)


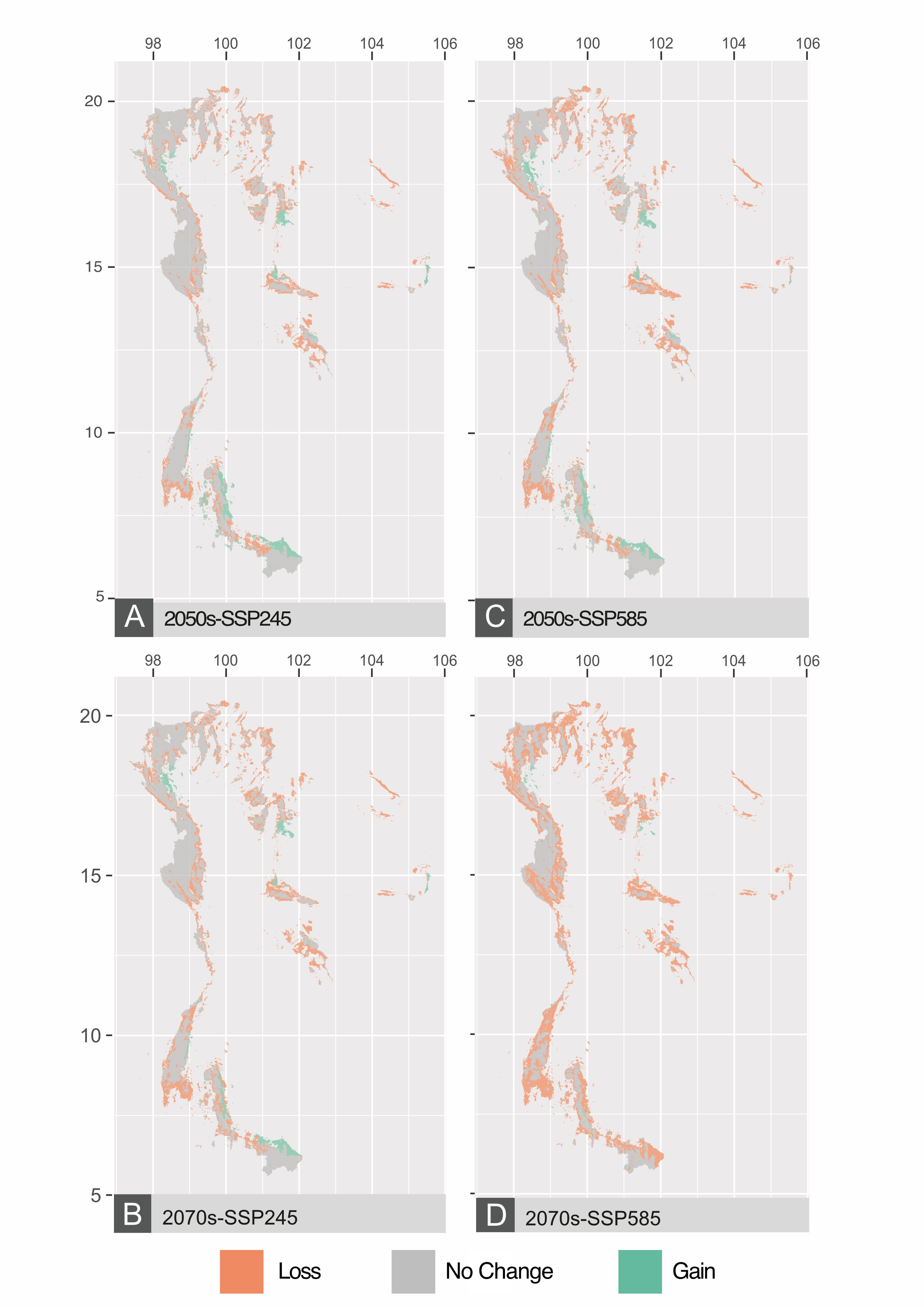


FIGURE S6 Projected distribution changes for *Begonia* species in Thailand under different periods and climate scenarios. The habitat suitability represented by three different colors based on a percentage of model agreement: lost (orange), unchanged (grey), or gained (green). The maps visualized the years 2050s and 2070s under two Shared Socioeconomic Pathway (SSP) scenarios: SSP245 (medium greenhouse gas emissions concentration) and SSP585 (high greenhouse gas emissions concentration).
